# Intrinsic mechanical behavior of femoral cortical bone in young, osteoporotic and bisphosphonate-treated individuals in low- and high energy fracture conditions

**DOI:** 10.1038/srep21072

**Published:** 2016-02-16

**Authors:** Elizabeth A. Zimmermann, Eric Schaible, Bernd Gludovatz, Felix N. Schmidt, Christoph Riedel, Matthias Krause, Eik Vettorazzi, Claire Acevedo, Michael Hahn, Klaus Püschel, Simon Tang, Michael Amling, Robert O. Ritchie, Björn Busse

**Affiliations:** 1 Department of Osteology and Biomechanics, University Medical Center Hamburg, D-22529 Hamburg, Germany; 2 Experimental Systems Group, Advanced Light Source, Berkeley, California, 94720, USA; 3 Materials Sciences Division, Lawrence Berkeley National Laboratory, Berkeley, California, 94720, USA; 4 Department of Medical Biometry and Epidemiology, University Medical Center Hamburg-Eppendorf, Martinistrasse 52, 20246 Hamburg, Germany; 5 Department of Forensic Medicine, University Medical Center Hamburg, D-22529 Hamburg, Germany; 6 Department of Orthopaedic Surgery, School of Medicine, Washington University, St Louis, MO 63110, USA; 7 Department of Materials Science and Engineering, University of California, Berkeley, California, 94720, USA

## Abstract

Bisphosphonates are a common treatment to reduce osteoporotic fractures. This treatment induces osseous structural and compositional changes accompanied by positive effects on osteoblasts and osteocytes. Here, we test the hypothesis that restored osseous cell behavior, which resembles characteristics of younger, healthy cortical bone, leads to improved bone quality. Microarchitecture and mechanical properties of young, treatment-naïve osteoporosis, and bisphosphonate-treated cases were investigated in femoral cortices. Tissue strength was measured using three-point bending. Collagen fibril-level deformation was assessed in non-traumatic and traumatic fracture states using synchrotron small-angle x-ray scattering (SAXS) at low and high strain rates. The lower modulus, strength and fibril deformation measured at low strain rates reflects susceptibility for osteoporotic low-energy fragility fractures. Independent of age, disease and treatment status, SAXS revealed reduced fibril plasticity at high strain rates, characteristic of traumatic fracture. The significantly reduced mechanical integrity in osteoporosis may originate from porosity and alterations to the intra/extrafibrillar structure, while the fibril deformation under treatment indicates improved nano-scale characteristics. In conclusion, losses in strength and fibril deformation at low strain rates correlate with the occurrence of fragility fractures in osteoporosis, while improvements in structural and mechanical properties following bisphosphonate treatment may foster resistance to fracture during physiological strain rates.

Like other bone diseases, osteoporosis is associated with increased risk of fracture^1^,^2^,^3^,^4^. Resistance to fracture is generated through deformation of bone’s multi length-scale elements (Fig. 1) and requires an optimal bone quality, which encompasses synergistic factors such as tissue composition (*e.g.*, collagen, mineral, crosslink profile, non-collageneous proteins), arrangement of structural features (*e.g.*, cortical porosity, Haversian canal density, trabecular architecture), and degree of damage (*e.g.*, microcrack density). However, some of the factors contributing to the bone’s characteristic fracture resistance become compromised with osteoporosis. These osteoporotic fractures most often affect the elderly population and occur at any skeletal location but most commonly the spine, hip, wrist, humerus and rib^5^,^6^. While bone fractures in healthy individuals occur in high energy traumatic events (*i.e.*, at high strain rates), in particular, osteoporotic bone fractures are additionally associated with fragility fractures occurring with minimal trauma (*i.e.*, at low strain rates)^7^,^8^,^9^,^10^.

To treat osteoporosis, a range of strategies have emerged that primarily target bone remodeling. One such treatment is a class of drugs called bisphosphonates, which act primarily by inhibiting bone resorption^11^,^12^. The reduced rate of fractures following bisphosphonate treatment has been attributed to structural and compositional reorganization of the bone tissue^13^,^14^,^15^,^16^,^17^ accompanied by restored osteoblastic and osteocytic cell characteristics counterbalancing the accumulation of micropetrosis^18^,^19^,^20^,^21^. However, the rare advent of atypical femoral fractures associated with long-term anti-resorptive treatment has led to further questions about bisphosphonate’s mode of action at femoral cortical sites and other skeletal sites^22^. Considering bone’s multi-scale structure generating strength (*i.e.*, resistance to plastic deformation) through inelastic deformation of the fibril nanostructure (*i.e.*, fibrillar sliding, sacrificial bonding)^23^,^24^,^25^,^26^, we aim to investigate whether bisphosphonate treatment alters the *intrinsic* fibrillar-level mechanical properties at relevant strain rates (*i.e.*, non-traumatic and traumatic fractures). Using femoral cortical bone from young, osteoporosis and bisphosphonate-treated cases, first we deconstruct how the bone microstructure and composition change with age, disease and treatment as well as the effects on the intrinsic mechanical behavior in non-traumatic (low energy) and traumatic (high energy) fractures. Therefore, high-resolution imaging techniques were used in combination with three point-bending and small-angle x-ray scattering (SAXS) to provide insight into bone quality in young, osteoporotic and bisphosphonate-treated cases with special emphasis on the characteristics of high-energy traumatic fractures and low-energy fragility fractures.

## Materials and Methods

Human femoral cortical bone was obtained at autopsy from the Department of Forensic Medicine at the University Medical Center, Hamburg, Germany. The fresh tissue was frozen after removal. Samples for mechanical testing were cut from the mid-diaphysis of the femur on the lateral side. The female cohort consisted of the following groups:

- *Young* (n = 5). Healthy individuals with no indication of bone disease
- *Osteoporosis cases* (n = 5). Individuals diagnosed with osteoporosis (osteodensitometry, medical records) but no documented history of bisphosphonate treatment
- *Bisphosphonate-treated cases* (n = 5). Individuals taking bisphosphonates (alendronate) for a duration of 6 years due to manifested osteoporosis.

The mean age of the individuals was 34.8 ± 4.8 years (mean age ± s.d.) for the young group, 80.2 ± 9.4 years for the osteoporosis group and 79.4 ± 7.9 years for the bisphosphonate-treated group. All individuals suffering from cancer, renal disease, primary hyperparathyroidism, and Paget’s disease of bone or showing any other circumstances that could lead to secondary bone disease (*i.e.*, immobilization or hospitalization) were excluded from the study. Informed consent was obtained from the family members after comprehensive information on all related issues. The study was approved by the Ethics Committee of the Hamburg Chamber of Physicians (PV3486) and the methods were carried out in accordance with the approved guidelines. The osteoporosis and bisphosphonate bone samples were acquired as part of the *ex vivo* BIOASSET consortium (biomechanically founded individualized osteoporosis assessment and treatment).

### Dual Energy X-ray Absorptiometry

T-Score was determined using DXA (Lunar Prodigy, Lunar Corporation, Madison, WI, USA) for the lumbar spine and both proximal femurs. Thus, we were able to categorize the osteoporosis and bisphosphonate-treated cases according to WHO criteria. Female individuals over the age of 65 with no history of osteoporosis treatment were included in the study if a post-mortem DXA score in the hip or spine was less than −2.5, which indicates osteoporosis. Additionally, female individuals were included in the study if previously diagnosed with osteoporosis and used alendronate for 1 or more years. The data presented here focuses on the difference between the osteoporosis and long-term bisphosphonate-treated individuals.

### High-resolution peripheral quantitative computer tomography (HR-pQCT)

HR-pQCT (Xtreme CT, Scanco Medical AG, Switzerland) was performed on the distal radius and distal tibia following standardized procedures^27^,^28^. A total of 104 slices were evaluated per site in each individual. Reconstruction with an isotropic voxel size of 82 μm, 512 × 512 matrix, was achieved with the Xtreme CT software^27^,^28^.

### Osteonal remodeling, osteocyte lacunar characteristics and structure indices

Quantitative backscattered electron imaging and histomorphometry on Toluidine blue stained sections were used to determine the osteonal microstructure and osteocyte lacunar characteristics in all study groups. The following parameters were directly measured: number of Haversian canals per bone area (#/mm^2^), number of mineralized lacunae per bone area (#/mm^2^) and Haversian area per tissue area (%). Staining of the specimens enabled static bone histomorphometry (Osteoquant, Bioquant Image Analysis Corp., Nashville, TN) in accordance with ASBMR (American Society of Bone and Mineral Research) guidelines^29^. The histomorphometric assessment of eroded surfaces per bone surfaces (ES/BS, %) was measured to provide bone resorption indices^30^,^31^.

### Strength tests

Three-point bending tests were performed according to ASTM D790 to measure the strength of the cortical bone samples^32^. Rectangular beam samples were prepared with a low speed saw from cortical bone taken at the mid-shaft. The length of the samples was parallel to the osteonal orientation and the long axis of the femur. The rectangular bending samples had an average thickness of 3.8 mm, a height of 1.2 mm, and a length greater than 20 mm. The samples were tested on a mechanical testing device (Z.2.5/TN1S, Zwick/Roell, Ulm, Germany) with a 200-N load cell and a custom-made three-point bending fixture. The bending fixture consists of a bottom support span that holds two pins at a span of 16 mm. The bending test was performed in displacement control at a displacement rate of 0.01 mm/s. The bending modulus was obtained by fitting the linear portion of the curve to a straight line, the maximum bending stress is defined as the maximum stress attained during testing, and the yield stress was obtained through the 0.2% offset method.

### Porosity measurements

Following the bending test, the bend samples were imaged using micro-computed tomography (Scanco Medical μCT 42, Brüttisellen, Switzerland) to measure the porosity. The scans were performed at 55 kV, 145 μA, with a voxel size of 10 μm and an integration time of 200 ms. After marking the volumes of interest and thresholding, the micro-architecture was automatically evaluated using the micro-CT evaluation program V6.5-2 with direct 3D bone morphometry.

### Small-angle x-ray scattering (SAXS)

The mechanical properties at the fibrillar length-scale were investigated via synchrotron small-angle x-ray scattering experiments at low and high strain rates. In these experiments, a tensile test was performed on a sample of bone while the sample was simultaneously exposed to x-rays^1^. As the ordered nano-level structure of the bone, specifically the 67-nm periodicity of the mineralized collagen fibril, diffracts the x-rays numerous SAXS measurements throughout a tensile test allowed the deformation at small length-scales to be measured. During the tensile testing experiments, measurements of the load, images of the sample surface (for tissue strain measurement), and 2D small-angle x-ray scattering patterns (for fibril strain measurement) were collected simultaneously. After data analysis, the fibril strain as a function of tissue strain can be deduced.

To prepare cortical bone samples for SAXS, slices of cortical tissue were cut to dimensions of 15 mm ×1 mm ×250 μm with a band saw. The samples were then fixed by soaking in 70% ethanol. The samples were subsequently rehydrated in Hanks’ Balanced Salt Solution (HBSS) and then air dried for 12 h. Next, silicon carbide paper was glued to the ends of the samples with cyanoacrylate glue to provide a surface to grip during mechanical tensile testing. Four tensile samples of cortical bone were prepared from each individual with the samples soaked in HBSS for 12 h prior to testing.

Test specimens were tested at two physiological strain rates^33^. Strain rates of 10^−2^ and 10^−5^ s^−1^ were produced with displacement rates of 1.0 and 0.001 mm/s, respectively, on a sample with a 10-mm distance between the grips. The faster strain rate corresponds to the physiological strain during running, while the slower strain rate corresponds to quasi-static loading^33^,^34^,^35^,^36^. For the low strain rates, the samples were loaded in tension in a custom-made mechanical testing device, while for the high strain rate tests, a mechanical testing device (TST350 tensile stage, Linkam Scientific Instruments, Surrey, UK) was used. The rig was positioned in beamline 7.3.3 at the Advanced Light Source (ALS) synchrotron radiation facility (Lawrence Berkeley National Laboratory, Berkeley, California, USA), such that SAXS data collection could be recorded simultaneously with mechanical loading^37^. For these experiments, a high-speed Pilatus 2 M detector (Dectris, Baden, Switzerland) was positioned ~4000 mm from the sample to collect SAXS data using an x-ray energy of 10 keV. During the low strain rate tensile test, data were collected for 0.5 s every 10 s. At high strain rates, a burst of images was acquired with an exposure time of 0.01 s every 0.0125 s.

The analysis software Igor Pro (Wavemetrics, Portland, Oregon, USA) was used in conjunction with the custom macro NIKA to calibrate the sample-to-detector distance and beam center from an x-ray exposure of a silver behenate standard sample^38^. Following calibration, custom software written in LabVIEW (National Instruments, Austin, Texas, USA) was used to transform all exposures to polar coordinates (maps of azimuthal angle *vs.* q). A weighted spline function was fit to the scattering curve at each azimuthal angle (*i.e.*, the area containing the first-order collagen peak being weighted lightly, and the remainder of the curve weighted heavily) and subtracted from the data to remove diffuse scattering near the beam center. Azimuthal peaks were then detected and fitted with Gaussian functions to locate the angle of orientation of the collagen. In each 2D dataset, diffraction from the collagen fibril produces two collagen peaks around the beam center separated by 180 degrees. Therefore, the 2D data were converted to a 1D scattering curve by integrating each collagen peak between an azimuthal angle of + /− 5 degrees of the collagen orientation angle and combining the results. The first-order peak from the collagen was then fitted to an exponentially modified Gaussian function, from which peak height, area, width (FWHM), and center location were measured. The strain in the mineralized collagen fibrils was measured as the change in position of the corresponding peak’s center divided by its location at zero load.

The tissue strain was measured by imaging the change in spacing of horizontal lines marked on the sample’s surface, which were later analyzed using a custom image analysis software utilizing the software package Vision Assistant 8.5 (National Instruments, Austin, Texas, USA). The displacement of the lines was divided by the separation at zero load to determine the bulk tissue strain.

### Fracture surface analysis

Following the SAXS analyses, at low and high strain rates the surfaces of the fractured samples were imaged with an opto-digital light microscope (DSX500i, Olympus, Japan). Samples were mounted on an inverted microscope with a motorized stage and scanned with an optic zoom.

### Advanced glycation end-products (AGEs)

A fluorometric assay was performed to evaluate the extent of AGEs in the cortical bone samples. A section of the femoral cortical bone was demineralized using EDTA (ethylenediamine tetraacetic acid) and then hydrolyzed using 6 N HCl (24 h, 110 °C). AGEs content was determined using fluorescence readings taken using a microplate reader at the excitation wavelength of 370 nm and emission wavelength of 440 nm. These readings were standardized to a quinine-sulfate standard and then normalized to the amount of collagen present in each bone sample. The amount of collagen for each sample was determined based on the amount of hydroxyproline, the latter being determined using a chloramine-T colorimetric assay that recorded the absorbance of the hydrolysates against a commercially available hydroxyproline standard at the wavelength of 585 nm.

### Statistics

Statistics were performed using SPSS 22 (IBM, Armonk, New York, USA). Results are reported as mean ± standard deviation. Normal distribution of the data was tested with the Shapiro-Wilk test. A one-way analysis of variance (ANOVA) was used to test for statistical significance. When significance was found, a Tukey post-hoc test was performed to determine differences between the groups. The significance level was set to α = 0.05. The mechanical properties derived by SAXS analyses were accessed with linear mixed models using a polynomial fit function, where we allowed varying intercepts and slopes between individuals. The significance level was set to α = 0.05

## Results

### Characteristics of the cases

#### Osteodensiometry

Dual-energy x-ray absorptiometry (DXA) was used for measuring the bone mineral density (BMD). Measurements at the spine level revealed a T-score = −3.24 ± 1.26 in the osteoporosis group in comparison to −1.50 ± 2.51 in the bisphosphonate treatment group, p = 0.22. At the hip level the DXA measurements in the osteoporosis group showed a T-score of −3.53 ± 1.02 vs. a T-score of −2.80 ± 0.97, p = 0.32) in the bisphosphonate treatment group. High resolution peripheral quantitative computer tomography (HR-pQCT): The trabecular bone density (Dtrab) of the study groups was assessed in the radius, where the osteoporosis group revealed 118.60 ± 17.61 mg HA/ccm in comparison to 68.45 ± 37.59 mg HA/ccm in the bisphosphonate treatment group (p = 0.07). 2D histomorphometry: The bisphosphonate treatment led to a reduction in bone resorption as measured through a 50% lower eroded surface (eroded surface / bone surface, ES/BS) in vertebral trabecular bone (L5) (ES/BS in the osteoporosis group = 12.2 ± 1.4 vs. 6.1 ± 1.6 in the bisphosphonate treatment group, p = 0.0005).

#### 3D structural indices

**Figure 2:**
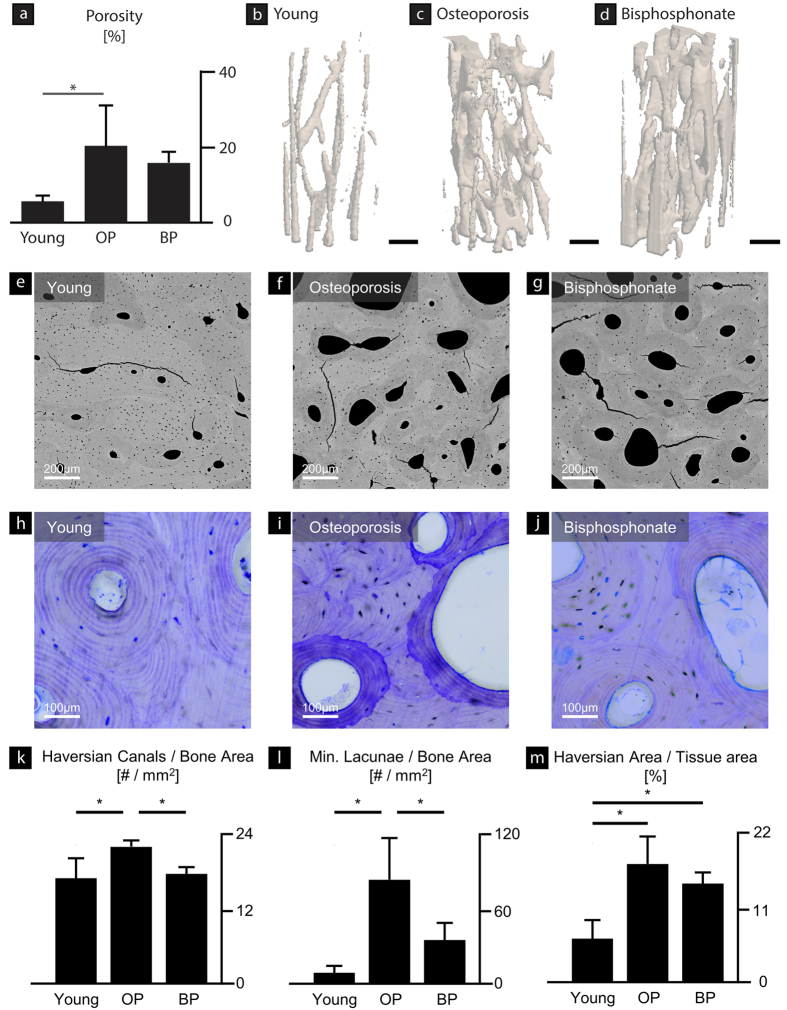
Microstructural characteristics. The porosity of the bending samples was measured with micro-computed tomography scans. (**a**) The young samples had a lower porosity than the osteoporosis (OP) and bisphosphonate-treated (BP) samples. The changes in porosity were significant between the young and osteoporosis cases (p = 0.01). Representative changes in porosity among the (**b**) young, (**c**) osteoporosis, and (**d**) bisphosphonate-treated cases are visible in three-dimensional images from the micro-computed tomography scans. Scale bars are 250 μm. (**e–g**) Backscattered scanning electron microscopy and (**h–j**) Toluidine-blue-stained histological sections show the differences in microstructural features for each case. (**k**) Indeed, the number of Haversian canals in the osteoporotic bone is 29% greater than the young (p = 0.004) and 24% greater than the bisphosphonate-treated cases (p = 0.010). (**i**) The number of mineralized lacunae was found to be significantly higher in the osteoporosis group in comparison the young (p < 0.001) and bisphosphonate-treated groups (p = 0.009). (**m**) The size of the Haversian area reflecting the 2D porosity was 2.7-fold greater in the osteoporotic tissue than the young (p < 0.001) and 2.3-fold greater in the bisphosphonate-treated group than in the young group (p = 0.002).

Micro-computed tomography measurements were used to measure the bone volume fraction in the bending samples. The porosity of the samples was lowest in the young cases followed by the bisphosphonate-treated cases and the osteoporosis cases (Fig. 2a), which can be seen in the three-dimensional reconstructions of the micro-computed tomography scans (Fig. 2b–d). However, the porosity values were only significantly different between the young and osteoporosis cases (p = 0.01), where the osteoporosis cases have 3.5-fold greater porosity.

#### 2D histomorphometry

The microstructural differences between the samples are illustrated in representative images acquired with quantitative backscattered electron imaging (Fig. 2e–g) and Toluidine-blue stained histological sections (Fig. 2h–j). Quantifications from the backscattered images of the number of Haversian canals per bone area, the number of mineralized lacunae per bone area and the Haversian area per tissue area are shown in Fig. 2k–m. The osteoporotic samples had a significantly higher number of Haversian canals than the young cases (p = 0.004), which indicates increased osteoclastic resorption. The number of mineralized lacunae per bone area showed significantly higher numbers in osteoporosis in comparison to young (p < 0.001) and bisphosphonate-treated bone (p = 0.009), which indicates increased osteocyte apoptosis in osteoporosis. Additionally, the trends in the Haversian canal area per bone area, which reflects the 2D porosity, were similar to the 3D cortical porosity measured with micro-computed tomography (Fig. 2a). Here, the Haversian canal area per bone area was significantly higher in the osteoporotic than in the young cases (p < 0.001).

#### Advanced glycation end-products (AGEs)

The content of AGE cross-links was measured in cortical bone samples from young, osteoporosis and bisphosphonate-treated cases. The results of the fluorometric assay did not show any significant differences in AGE content between the groups. The average value for AGEs content (measured in μg Quinine / g Collagen) was 6.52 ± 2.46 for the young cases, 7.91 ± 2.54 for the osteoporosis group, and 5.85 ± 1.39 for the bisphosphonate-treated osteoporosis group.

### Mechanical properties

#### Elastic and inelastic mechanical properties from strength tests

**Figure 3:**
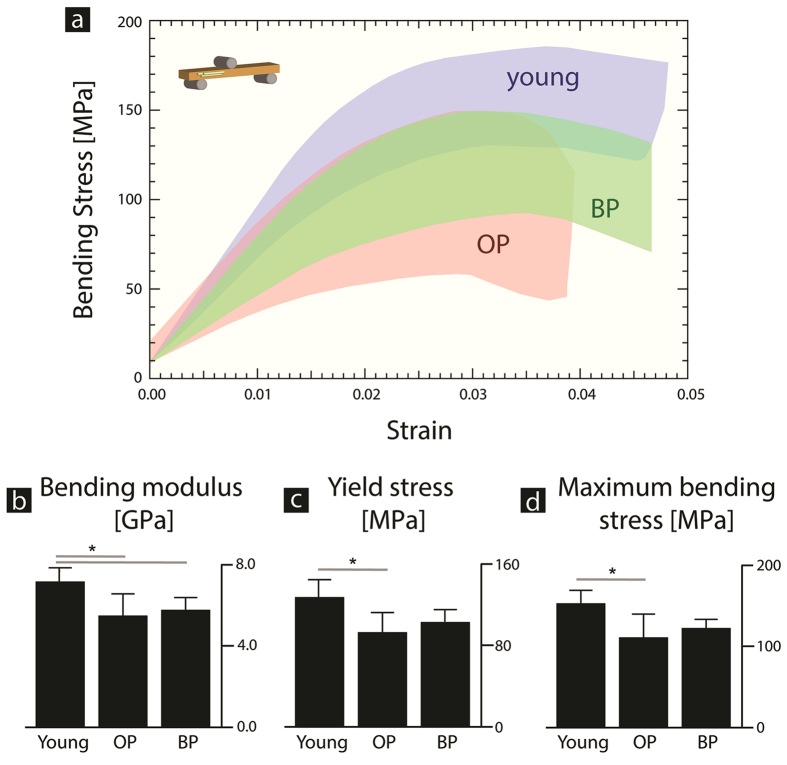
Cortical bone strength with bisphosphonates. Human cortical bone samples from the mid-diaphysis of the femur were tested in three-point bending to measure the strength. (**a**) The stress-strain curves are shown for the young, osteoporosis (OP) and bisphosphonate-treated (BP) cases. Here, the shaded area contains all of the stress-strain curves for each group. (**b**) The bending modulus of the OP (p = 0.02) and BP (p = 0.04) groups were both significantly lower than in the young cases, which may be due to the lower mineralization values previously reported for the same cases^21^. (**c**) The yield stress and (**d**) maximum bending stress were also both significantly lower in the OP cases (p = 0.02).

The strength of the human cortical bone samples was measured via three-point bending of unnotched beams. The results of the strength tests are shown in Fig. 3a, where the shaded area shows the boundary of all stress-strain curves for that study group. From the stress-strain curves, the bending modulus (*i.e.*, measuring the resistance to elastic deformation) is 22% lower for the osteoporosis cases (p = 0.02) and 18% lower for the bisphosphonate-treated cases (p = 0.04), in comparison to the young group (Fig. 3b). Additionally, the maximum bending stress and the yield stress (*i.e.*, measuring the resistance to plasticity) both decreased by 27% (p = 0.02 in both cases) in the osteoporosis cases, in comparison to the young group (Fig. 3c,d). In general, the results suggest that the osteoporosis cases have a lower resistance to plasticity in comparison to the young cases.

#### Fibril strain at low strain rates

**Figure 4:**
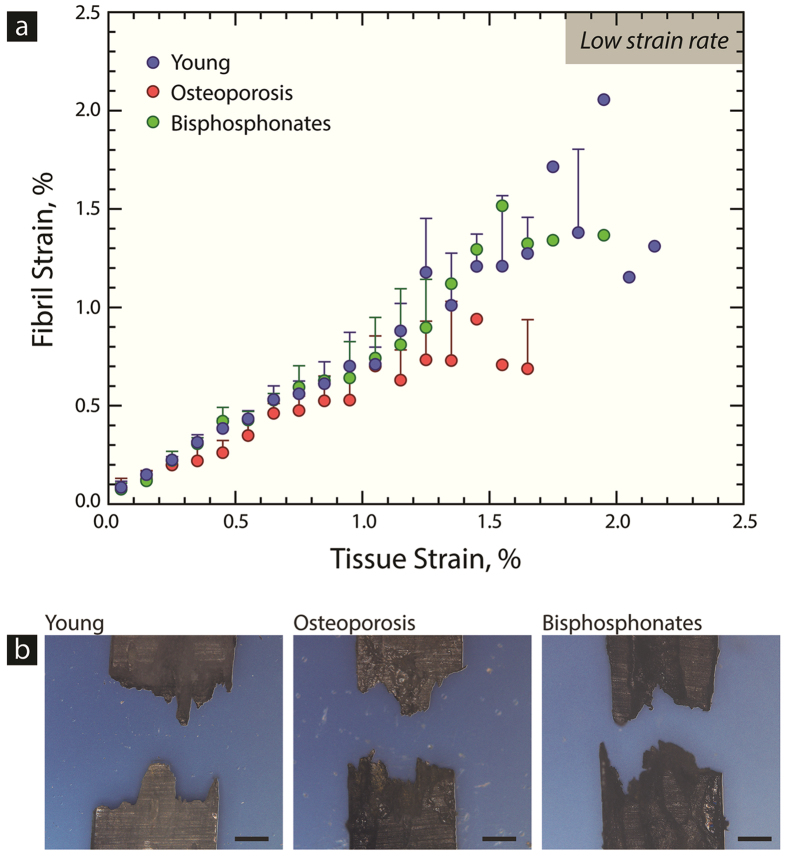
Small angle x-ray scattering (SAXS) of bisphosphonate-treated human cortical bone. Human cortical bone samples from the mid-diaphysis of the femur were mechanically tested in tension at a low strain rate, while simultaneously using synchrotron SAXS to discern the fibril-level deformation in the young, osteoporosis, and bisphosphonate-treated osteoporosis cases. (**a**) The strain in the fibril during the mechanical tension test is measured as a function of the strain applied to the whole sample (*i.e.*, the tissue strain). Essentially, the material behavior indicates elastic stretching of the fibril during the initial linear portion of the curve. After the linear region, inelastic deformation begins to occur, which can result in more heterogeneous behavior. However, a plateau in the curve, where a steady-state fibril strain is reached, may indicate that fibrils are slipping past one another while the tissue continues to stretch (*i.e.*, fibrillar sliding). The osteoporosis cases have a more pronounced plateau in the fibril strain (p < 0.001), indicating lower fibril deformation than the young cases, which has been similarly seen in aging and could lead to earlier failure^1^. In contrast, the bisphosphonate-treated cases exhibit more fibrillar deformation, which corresponds to the behavior of the young cases, and may explain the improvements in strength. (**b**) A profile view of the tensile samples shows the roughness of the fracture surfaces for each case. At low strain rates, the samples all exhibit rough fracture surfaces, which is characteristic of the generation of plasticity at low strain rates. Scale bar is 250 μm.

Synchrotron SAXS measurements were used to investigate deformation of the mineralized collagen fibrils during low strain rate tensile tests in the young, osteoporosis and bisphosphonate-treated groups. Here, the strain in the fibrils was measured as a function of tissue strain (*i.e.*, strain applied to sample), as shown in Fig. 4a. In the beginning of the curves, the strain in the fibrils linearly increases with tissue strain, which represents stretching of the fibrils in the elastic region. As the sample begins to inelastically deform, the fibril strain becomes more heterogeneous and can reach a plateau (*i.e.*, a steady-state fibril strain), which has been interpreted as the phenomenon of fibril sliding^23^. During fibrillar sliding, the fibrils maintain a constant strain and further increases in tissue strain occur due to sliding between the fibrils. A profile view of the SAXS samples (Fig. 4b) shows rough fracture surfaces characteristic of energy absorption and plasticity.

The significance of the SAXS experiments was assessed with a linear mixed model that estimated significant differences between the study groups. Specifically, the second-order term for the osteoporosis group that describes the plateau in the fibril strain is estimated to be significant (p < 0.001).

Altogether, the SAXS results in Fig. 4a indicate that the fibrils in the osteoporosis samples have an altered response to mechanical deformation. Clearly, at a given tissue strain, the fibrils are deforming less in the osteoporotic tissue than the young tissue. Conversely, the higher fibril strains in the bisphosphonate-treated samples may indicate that the bisphosphonate treatment improves nano-level aspects of the structure that facilitate plasticity generation in bone tissue.

#### Fibril strain at high strain rates

**Figure 5:**
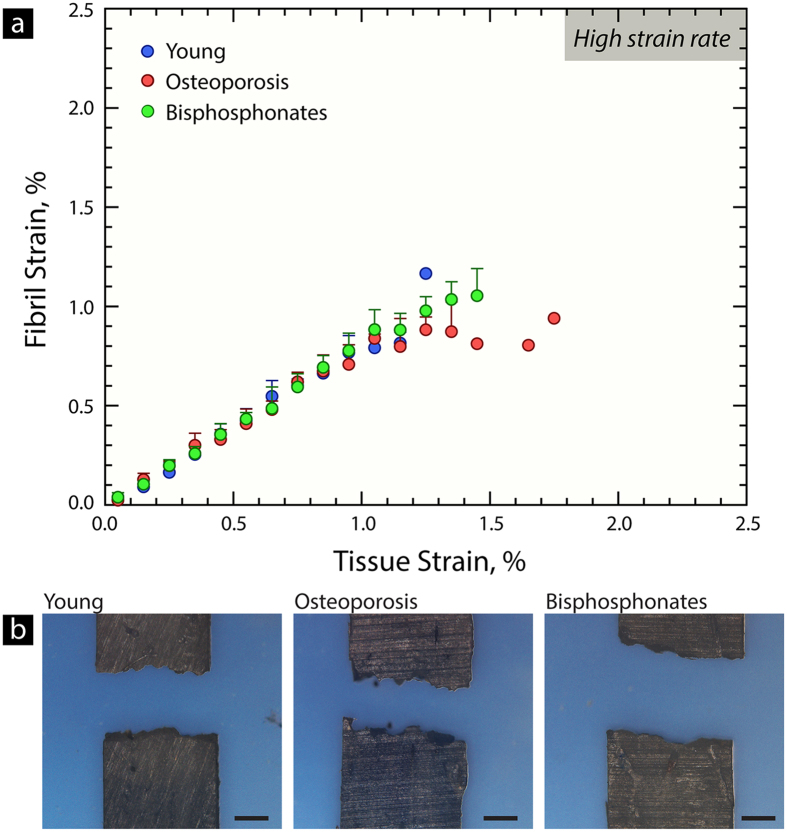
Small angle x-ray scattering (SAXS) at high strain rates. Human femoral cortical bone samples were also mechanically tested in tension at a high strain rate, while using synchrotron SAXS to discern the fibril-level deformation. The fibril strain can be measured as a function of the strain applied to the whole sample (*i.e.*, the tissue strain). (**a**) At high strain rates, the fibril versus tissue strain is very linear, which indicates that the plasticity sliding mechanisms are “locking up” and deformation is dominated by elastic stretching of the fibril. Similar behavior has been found for young bone at high strain rates^33^. Indeed, plasticity in human cortical bone most likely derives from the viscoelasticity of the fibril structure, which enables fibrillar sliding and sacrificial bonding mechanisms to absorb energy^33^,^39^. Higher strain rates “lock up” the plasticity mechanisms at the fibril level. (**b**) The profile of the tensile samples shows that the fracture surfaces have a lower roughness at high strain rates, which corresponds with the lower levels of plasticity measured during the SAXS tests. Scale bar is 250 μm.

Synchrotron SAXS measurements were used to measure fibril-level deformation during high strain rate tensile tests in the young, osteoporosis and bisphosphonate-treated groups. Here, the fibril strain is plotted as a function of tissue strain (*i.e.*, strain applied to sample) in Fig. 5a. The curves at high strain rates are generally linear, which is similar to previous results, and indicates less plasticity than at low strain rates^33^,^39^. Additionally, the fibril and tissue strains were smaller at higher strain rates in comparison to low strain rates. A profile view of the SAXS samples tested at high strain rates (Fig. 5b) shows a flatter fracture surface indicative of lower levels of plasticity.

## Discussion

The mechanical integrity of bone tissue is a result of the composition, deformation and fracture of its hierarchical bone structure: therefore, the quality of this complex structure is of upmost importance^26^,^40^. Osteoporosis is associated with changes in bone quality that increase bone fracture risk, primarily increasing susceptibility to fragility fractures that occur with minimal trauma. Bisphosphonates are commonly used to treat osteoporosis and have been found to reduce fracture risk, particularly at trabecular sites. Due to the association of long-term bisphosphonate use with atypical femur fractures and questions regarding the effects of bisphosphonates on cortical tissue, here, we unraveled changes in structure and mechanical properties of young, osteoporosis and bisphosphonate-treated bone in the femoral cortex. Currently, very few studies have characterized changes in cortical bone quality (*i.e.*, structure and mechanical properties) in humans with clinically diagnosed osteoporosis in comparison to bisphosphonate-treated cases^21^,^41^,^42^,^43^,^44^,^45^. While many clinical studies have shown that bisphosphonate treatments are associated with reduced fracture risk in osteoporotic patients, the osseous characteristics have been primarily assessed as bone volume fractions at trabecular sites^13^,^14^,^15^,^16^,^17^,^46^. However, the characteristics of trabecular sites may not be directly transferable to cortical regions because bisphosphonates have been reported to cause different effects at different skeletal sites^21^,^47^. The reason for the site-specificity may be that the surface-area-to-volume ratio is much higher in porous trabecular bone than dense cortical regions, which may allow more accumulation of the drug in trabecular sites^48^.

The results of the performed strength tests (Fig. 3a) provide a measure of the elastic and inelastic properties of the cortical bone. Here, the modulus was found to be significantly lower in the case of the osteoporotic and bisphosphonate-treated cases (Fig. 3b). A previous study on the same set of cases reported a significantly higher mineralization distribution in the cortical bone of young cases than in both osteoporotic and bisphosphonate-treated cases, which was measured via quantitative backscattered electron imaging^21^. The lower level of cortical mineralization in the osteoporosis and bisphosphonate-treated groups could be attributed to changes in bone turnover. Similarly, a previous study by Donnelly *et al.* using biopsies from the proximal femur, found no difference in mineral content between bisphosphonate-naïve and bisphosphonate-treated groups^42^. Thus, the lower modulus or resistance to elastic deformation in the osteoporosis and bisphosphonate-treated cases is most likely a result of the lower mineral content^49^. At smaller length-scales, osteoporotic tissue has lower mineralization densities in the femoral cortical bone, measured via quantitative back-scattered electron imaging^21^,^50^.

The stress-strain curves from the three-point bending tests (Fig. 3a) also provide a measure of the bone’s inelasticity. Here, the osteoporotic cases have a significantly lower yield stress and maximum bending stress (Fig. 3c,d). However, no significant differences were detected in the bisphosphonate-treated group. The origins of these changes in the bone’s inelasticity due to osteoporosis and possibly bisphosphonate treatment originate due to changes in bone mass and matrix characteristics.

Osteoporosis is associated with losses in cortical bone quantity, primarily through trabecularization of endosteal surfaces producing cortical porosity^50^,^51^. Here, higher levels of cortical bone loss and porosity in post-menopausal women correlate with a higher risk of bone fracture^50^,^52^. At the microstructural scale, significantly smaller osteon diameters and fewer osteocyte lacunae have been reported in the proximal femur as well as higher Haversian canal densities and numbers of mineralized lacunae^21^,^43^. Differences in bone volume fraction were measured between the three groups with micro-computed tomography and 2D bone morphometry of the samples (Fig. 2). The young group had a significantly lower porosity than the osteoporosis cases, while the differences between the young and bisphosphonate-treated cases reflected only minor differences in porosity. At other skeletal sites, especially trabecular regions, bisphosphonates are associated with increases in bone mass (*i.e.*, BV/TV)^13^,^14^,^15^,^16^,^17^,^44^. However, due to the skeletal variations in the effects of bisphosphonates, reductions in porosity may not occur in predominantly cortical regions^21^. A number of studies investigating cortical porosity in the distal radius or tibia following alendronate treatment have found that the cortical porosity did not necessarily change in comparison to treatment-naïve controls, especially after longer use^53^,^54^.

Strength is generated from nano-level inelastic deformation, principally at the fibril level (*i.e.*, fibrillar sliding, sacrificial bonding)^23^,^24^,^25^,^26^. SAXS experiments were used to assess the fibril level deformation and to determine whether changes in matrix quality contributed to the reduced strength found in the osteoporotic cases. At low strain rates (*i.e.*, quasi-static loading conditions), the fibril strain reaches a plateau in the osteoporosis cases (p < 0.001), where fibril deformation is lower than in the young cases (Fig. 4), which could explain why the tissue has a lower strength and is more susceptible to fragility fractures. For the osteoporosis cases to have a lower amount of fibril strain, the structure in osteoporotic tissue must be restricting deformation, which is similar to the effects of aging, where higher levels of cross-linking restrict fibrillar strain before sliding^1^ The mechanism restricting fibril deformation must originate from osteoporosis-related changes in the nano-level structure, such as enzymatic or non-enzymatic cross-linking, collagen quality (*i.e.*, post-translational modifications to the collagen) or non-collagenous proteins. In the nanostructure, non-enzymatic cross-links have been shown to play a large role in aging, where cross-links increase with age and restrict fibril deformation^1^,^55^. However, here, the results of the fluorometric assay did not uncover significant differences in the quantity of non-enzymatic AGE cross-links, perhaps due to changes in bone turnover associated with osteoporosis that limit the accumulation of AGEs^56^. Previous studies investigating AGE content with alendronate treatment in a canine model found that the AGE content did not change at a dose level comparable to that used for the treatment of osteoporosis in post-menopausal women^57^. However, alterations to the enzymatic and non-enzymatic cross-linking profiles have also been reported, between fracture and non-fracture cohorts in women^58^,^59^.

While AGEs may not be the source restricting fibrillar sliding, osteoporosis has been associated with post-translational modifications to the collagen apart from cross-linking (*e.g.*, hydroxylation of lysine) and changes in non-collagenous proteins, which could affect deformation of the fibril^60^,^61^,^62^,^63^,^64^,^65^. Thus, the osteoporotic bone has reduced fibril deformation, while the fibrils in the bisphosphonate-treated cases tend to have a behavior comparable to young bone (Fig. 4a). Therefore, the bisphosphonate treatment may be improving the quality of the tissue at the nano-level. Indeed, previous studies have shown that changes of the osteoblast and osteocyte characteristics following bisphosphonate treatment can improve the quality of the collagen and bone tissue produced during bone formation^21^,^66^.

Additionally, the SAXS measurements were performed at high strain rates, which is physiologically comparable to the rate of loading during walking, running or traumatic events. At high strain rates, cortical bone has less plasticity^33^,^67^. The time-dependent deformation of human cortical bone most likely results not from the viscoelasticity of collagen but deformation in and between fibrils^33^,^39^. At high strain rates, the plasticity mechanisms responsible for fibrillar sliding or sacrificial bonding “lock up”, and fibril deformation is dominated by elastic stretching^33^. Thus, in Fig. 5, it is not surprising that the fibril *vs.* tissue strain has a linear slope at high strain rates for the young cases. The osteoporosis cases have a similar behavior but there is a slight trend towards lower strains (generally representing susceptibility to fracture).

Overall, our investigation of bisphosphonate-treated osteoporotic human cortical bone in patients indicates that administration of third generation bisphosphonates may lead to improvements in bone structure and function. However, cortical bone tissue in traumatic high strain rate conditions is susceptible to fracture in all cases. Thus, the viscoelastic behavior of the nano- and microstructural mechanisms resisting deformation and fracture “locks up” at high strain rates and reduces plasticity, which is necessary to generate toughness. Conversely, at low strain rates, healthy cortical tissue generates plasticity through stretching and sliding mechanisms. While these fibril deformation mechanisms are still active in osteoporotic tissue, the amount of fibril deformation is reduced, which may point to the origins of non-traumatic fragility fractures in osteoporotic patients. In general, the bisphosphonate-treated group reflects trends towards improvements in the nano-mechanical behavior in addition to advances in the previously reported microstructural characteristics, such as lower Haversian canal densities and fewer mineralized lacunae^21^. Future studies are required to examine the further changes to the nanostructure that are responsible for improved mechanical behavior after bisphosphonate treatment. However, our results show that administration of bisphosphonates leads to trends toward improving the mechanical properties in comparison to untreated osteoporotic cortical bone, which is in line with clinical studies of fracture incidence after bisphosphonate treatment^13^,^14^.

This study contains a few limitations. First, the data were collected from a rather small cohort of donors (n = 5 per group), which could pose a limitation due to the inherent variability in human cases. Beyond the assessed mechanical behavior in the young group, an additional aged-matched control group may provide additional insight. However, as this study focuses primarily on the effects of bisphosphonate treatment on cortical bone’s mechanical properties, which have been previously shown to restore osseous cell function by reducing the amount of mineralized osteocyte lacunae^21^, our data clearly supports improvement of bone quality and resistance to fracture following bisphosphonate treatment during physiological strain rates.

## Conclusions

Here, we analyzed the effects of third generation bisphosphonates (*i.e.*, six-year treatment with alendronate) on the structure and mechanical properties of cortical bone from the mid-diaphysis of the femur using a cohort of young, osteoporosis and bisphosphonate-treated cases. We conclude the following points:

- A lower elastic modulus was measured in the osteoporosis and bisphosphonate-treated cases in comparison to the young cases, which for these individuals was associated with a lower mineral content.
- Osteoporosis cases have significantly less resistance to plasticity and a lower strength, as measured by three-point bending strength tests.
- While all groups were susceptible to reduced plasticity at high strain rates representing traumatic conditions, the osteoporosis group was more susceptible to reduced fibrillar deformation at low strain rates, which could provide a mechanistic origin for osteoporotic fragility fractures.
- The osteoporosis group had a significantly higher porosity than the young group, which could contribute to lower bone strength.
- Changes in bone matrix quality with osteoporosis may also contribute to the lower bone strength. The osteoporosis group had lower fibrillar deformation, as measured through synchrotron small-angle x-ray scattering (SAXS). The lower fibrillar deformation could occur due to changes in the nano-level structure. Changes in the non-enzymatic cross-linking profile were not detectable here, but post-translational modifications to the collagen or alterations in the non-collagenous matrix have been associated with osteoporosis and could be responsible for the reduced plasticity.
- Bisphosphonate-treated tissue from cortical bone samples showed trends toward improved resistance to plasticity in strength and SAXS tests.

## Additional Information

**How to cite this article**: Zimmermann, E. A. *et al.* Intrinsic mechanical behavior of femoral cortical bone in young, osteoporotic and bisphosphonate-treated individuals in low- and high energy fracture conditions. *Sci. Rep.* **6**, 21072; doi: 10.1038/srep21072 (2016).

## Figures and Tables

**Figure 1:**
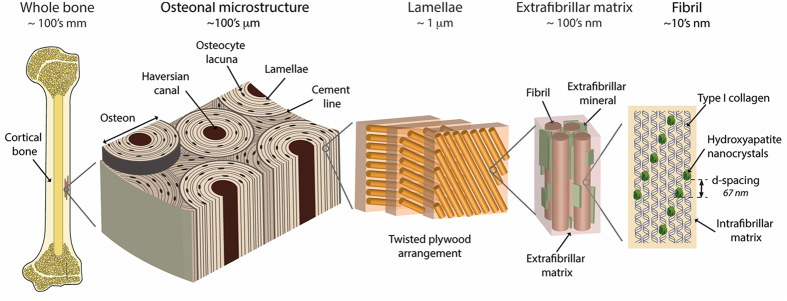
Structure of human cortical bone. Human bone comes in the form of either a porous trabecular framework or a dense cortical structure. Here, we focus on the cortical tissue, which can be found in the mid-diaphysis of the femur. In cortical bone, the microstructure consists of osteons (170–250 μm diameter)^43^, which are the units of bone produced during remodeling. The osteons contain a central vascular canal called the Haversian canal (60–90 μm diameter) that is concentrically surrounded by lamellae (2–9 μm thickness)^43^. The lamellae have a twisted plywood arrangement, where neighboring lamellae have different fibril orientations. Here, osteocyte cells reside in lacunae (15–25 μm diameter) that interconnect through canaliculi (100–400 nm in diameter). The lamellae are composed of collagen fibrils (80–100 nm diameter)^40^. The fibrils are surrounded by polycrystalline extrafibrillar mineral platelets. In addition to mineral, the extrafibrillar as well as the intrafibrillar matrix contains molecular components, such as non-collageneous proteins or cross-links, promoting the formation of sacrificial bonds^24^,^25^,^64^,^68^,^69^. Within the fibrils, type I collagen molecules (1.5 nm diameter, 300 nm length) and hydroxyapatite nanocrystals (50 nm width, 25 nm height, 1.5–4 nm thickness) form a composite structure, where arrays of collagen molecules staggered at 67 nm are embedded with nano-platelets of hydroxyapatite mineral. Adapted from Zimmermann *et al.*^70^.
